# Atmospheric seasonal forecasts of the twentieth century: multi‐decadal variability in predictive skill of the winter North Atlantic Oscillation (NAO) and their potential value for extreme event attribution

**DOI:** 10.1002/qj.2976

**Published:** 2017-02-01

**Authors:** Antje Weisheimer, Nathalie Schaller, Christopher O'Reilly, David A. MacLeod, Tim Palmer

**Affiliations:** ^1^ Department of Physics, National Centre for Atmospheric Science (NCAS) University of Oxford UK; ^2^ Research Department European Centre for Medium‐Range Weather Forecasts (ECMWF) Reading UK; ^3^ Department of Physics University of Oxford UK

**Keywords:** seasonal forecasting, extreme event attribution, skill of NAO predictions

## Abstract

Based on skill estimates from hindcasts made over the last couple of decades, recent studies have suggested that considerable success has been achieved in forecasting winter climate anomalies over the Euro‐Atlantic area using current‐generation dynamical forecast models. However, previous‐generation models had shown that forecasts of winter climate anomalies in the 1960s and 1970s were less successful than forecasts of the 1980s and 1990s. Given that the more recent decades have been dominated by the North Atlantic Oscillation (NAO) in its positive phase, it is important to know whether the performance of current models would be similarly skilful when tested over periods of a predominantly negative NAO. To this end, a new ensemble of atmospheric seasonal hindcasts covering the period 1900–2009 has been created, providing a unique tool to explore many aspects of atmospheric seasonal climate prediction. In this study we focus on two of these: multi‐decadal variability in predicting the winter NAO, and the potential value of the long seasonal hindcast datasets for the emerging science of probabilistic event attribution. The existence of relatively low skill levels during the period 1950s–1970s has been confirmed in the new dataset. The skill of the NAO forecasts is larger, however, in earlier and later periods. Whilst these inter‐decadal differences in skill are, by themselves, only marginally statistically significant, the variations in skill strongly co‐vary with statistics of the general circulation itself suggesting that such differences are indeed physically based. The mid‐century period of low forecast skill coincides with a negative NAO phase but the relationship between the NAO phase/amplitude and forecast skill is more complex than linear. Finally, we show how seasonal forecast reliability can be of importance for increasing confidence in statements of causes of extreme weather and climate events, including effects of anthropogenic climate change.

## 1 Introduction

Forecasts of seasonal‐mean anomalies of the climate using physically based circulation models are now routinely made at many operational meteorological forecast centres around the world. Such seasonal predictions provide estimates of seasonal‐mean statistics of weather, typically up to 6 months ahead. The physical basis for extended‐range predictions originates from slow variations in the lower boundary forcing of the atmosphere due to the dynamics of the oceans and the hydrology of the land masses, and from large‐scale components of the atmospheric general circulation with an intrinsic predictability time beyond that of individual synoptic weather systems, including the stratosphere (e.g. Palmer and Anderson, 1994; Sigmond *et al*., 2013).

The dominant mode of interannual variability of the coupled atmosphere–ocean system, the El Niño Southern Oscillation (ENSO), is a source of considerable seasonal predictability of the large‐scale atmosphere in the Tropics (Barnston *et al*., 2012) and, through global teleconnection patterns, also elsewhere in the world albeit to a lesser degree (Trenberth *et al*., 1998). Predicting the extratropical weather and climate is more difficult because atmospheric and oceanic instabilities and nonlinearities result in increased levels of variability compared to the Tropics. On the other hand, a large component of extratropical predictability is of tropical origin with circulation patterns in the Tropics influencing the extratropical circulation through teleconnections induced by Rossby wave dynamics (Hoskins and Karoly, 1981; Simmons, 1982; Greatbatch and Jung, 2007; Yu and Lin, 2016).

The exact extent to which these links between the Tropics and the extratropics translate into useful forecast information in seasonal predictions, however, remains an active area of research. In particular, the question of whether the North Atlantic Oscillation (NAO) and associated climate anomalies over the North Atlantic–European area during winter can be predicted with any confidence is still a matter of ongoing scientific debate. Müller *et al*. (2005) and Shi *et al*. (2015) reported that the skill of predicting the NAO in retrospective forecast experiments of previous years (also referred to as re‐forecasts or hindcasts) with quasi‐independent seasonal forecast models varies considerably over the last four decades. They found positive skill in predicting the interannual fluctuations in the atmospheric flow for more recent decades but in general not for hindcast periods started in the 1960s and 1970s. The study by Scaife *et al*. (2014) demonstrates high predictability of the NAO in the UK Met Office seasonal forecasting system over the 20‐year period 1992–2011. More recently, Dunstone *et al*. (2016) showed that the Met Office Decadal Prediction System produces similarly skilful NAO hindcasts (from 1981 onwards) and appears to have skill also in the second winter. However, the relatively short length of this hindcast set raises questions over the robustness of the skill estimates if tested during a different climate period. The Bayesian inference study by Siegert *et al*. (2016) suggests a high chance of a decrease in correlation skill if the hindcasts of Scaife *et al*. (2014) were evaluated over different periods. They further concluded that the particular 20‐year period was unusual and produced higher‐than‐normal correlation skill. Thus, an important open question with implication for future skill estimates is whether current‐generation models would be able to skilfully predict the NAO in earlier decades, e.g. around the 1960s where the models analysed by Müller *et al*. (2005) and Shi *et al*. (2015) struggled the most. Related questions include whether or not the variation of skill is monotonic in time, with further reduction in skill for even earlier decades, and if so what are the potential drivers?

In order to address these questions, we have performed a long retrospective atmospheric seasonal forecast experiment for all boreal winter seasons over the period 1900–2010. While decadal retrospective forecasts covering a similar period were presented in a recent study by Müller *et al*. (2014), the focus of their analysis was on decadal prediction skill of surface temperature. Here we concentrate on analysing one of the dominant modes of atmospheric variability in the extratropics, the NAO, and demonstrate how seasonal forecasts could prove useful for the emerging scientific area of probabilistic extreme weather and climate event attribution.

The aim of our article is threefold. Firstly, we present a new global atmospheric seasonal forecast dataset, called ASF‐20C, which covers the 110‐year re‐forecast period from 1900 to 2010 and consists of 51 ensemble members. The unprecedented size of the seasonal hindcast, both in terms of its length as well as its ensemble size allows for a thorough inspection of the robustness of forecast skill estimates and their variability on a centennial time‐scale. Secondly, we demonstrate that the ASF‐20C hindcasts indicate some multi‐decadal variability in predictive skill of the NAO, even though the uncertainty ranges around statistical skill estimates are necessarily large (Kumar, 2009). Our findings underline the importance of a representative re‐forecast dataset for robust conclusions about the levels of model skill in predicting the Atlantic–European climate in the future. Finally, we propose the use of our long ASF‐20C seasonal hindcast ensemble to complement attribution statements about extreme seasonal climate events with quantitative estimates of forecast reliability. Model‐based probabilistic event attribution can provide answers to the question of whether human activity increased the risk of occurrence of such events (for recent reviews of the subject, see Shepherd (2016) and Stott *et al*. (2016)).

The article is structured as follows. The next section describes the century‐long seasonal re‐forecast dataset. In section 3 we discuss the multi‐decadal variability of NAO forecast skill, followed by section 4 on the potential use of the century‐long hindcasts for event attribution studies. We finish with a summary and concluding remarks.

## 2 Atmospheric seasonal re‐forecasts of the twentieth century

Due to a lack of global sub‐surface ocean data to initialise the model in the first half of the century, the seasonal re‐forecasts have been carried out with an atmosphere‐only model using prescribed observed sea‐surface temperatures (SSTs) as lower boundary. Such a set‐up, which was widely used in the early days of dynamical seasonal prediction in the late 1990s (Palmer and Shukla, 2000; Shukla *et al*., 2000), can be seen as an experimental idealised version of the more complex coupled ocean–atmosphere seasonal forecasts. It assumes a perfect forcing of the atmosphere from the SSTs below and neglects any feedbacks from the atmosphere onto the SSTs. In particular, the oceanic ENSO forcing from the tropical Pacific SSTs is therefore prescribed, rather than predicted. However, the dynamical processes linking tropical and extratropical regions of the atmosphere are, in principle, represented in atmosphere‐only models and do not necessarily rely on an interactive coupling to the ocean. The NAO in particular is an internal mode of variability of the atmospheric circulation and the dynamical coupling with the ocean is not considered an essential feature of its dynamics (Greatbatch, 2000).

The atmospheric model used for the ASF‐20C re‐forecasts is version CY41R1 of the atmospheric component of the European Centre for Medium‐range Weather Forecasts (ECMWF)'s Integrated Forecasting System model (IFS). An earlier version of the model (CY36R4) coupled to an ocean model is used for the production of ECMWF's operational seasonal forecasting System 4 (Molteni *et al*., 2011). The horizontal spectral resolution of the model of T255 corresponds to a grid length of approximately 80 km with 91 vertical levels and the model top is at 0.01 hPa. The hindcasts were performed using the European atmospheric Re‐Analysis of the 20th Century (ERA‐20C: Poli *et al*., 2013, 2015) for initialisation and verification. ERA‐20C assimilates only surface pressure and marine wind observations. SSTs from the HadISST2.1.0.0 dataset (Rayner *et al*., 2003) were used to initialize and force the lower boundary. Seasonal re‐forecasts over 4 months were initialised for every 1 November during the period 1900–2009 which enables us to analyse the traditional boreal winter season of December to February (DJF) with a 2–4 month lead time. The DJF seasons are labelled according to the year the forecasts were initialised, that is, the DJF 1900 forecast corresponds to the forecasts initialised on 1 November 1900 and runs until the end of February 1901.

The re‐forecast experiments were set up in a way to mimic the operational System 4 (except for prescribed SST forcing and without singular vector perturbations on the initial state) as much as possible to enable a fair comparison with a forecasting system when only information before the initial date is available to use. In particular, this means that time‐varying greenhouse gas forcings were specified to improve the simulations of trends during the re‐forecast period. The forcings also include a time‐varying solar cycle and volcanic aerosols (Molteni *et al*., 2011).

The atmospheric forecast model includes an explicit stochastic representation of the uncertainties related to physical parametrizations of subgrid‐scale atmospheric processes, which generates an ensemble of 51 model realisations during the forecast runs. Weisheimer *et al*. (2014) showed that stochastic perturbations decrease some of the model biases in tropical deep convection and consequently improve ENSO forecasts.

**Figure 1:**
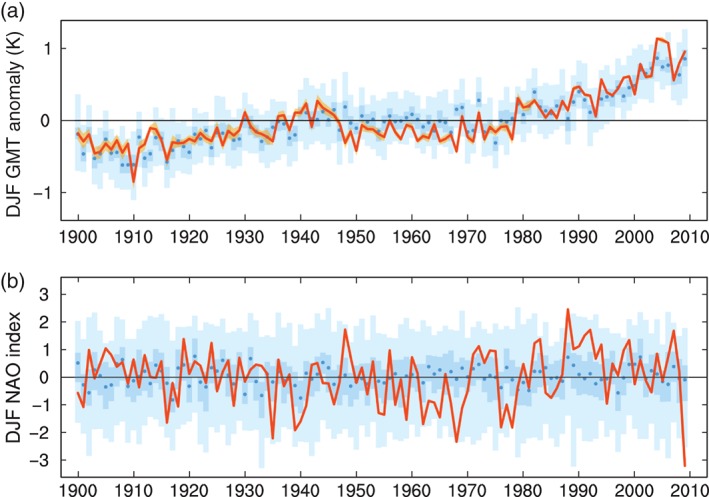
(a) DJF global mean 2 m temperature anomalies in ERA‐20C (red) and the re‐forecast ensemble of ASF‐20C (blue). Uncertainty estimates from the reanalysis and the re‐forecast ensemble are shown in orange (full range of the 10‐member ensemble) and with blue shades (light blue: full range; darker blue: interquartile 25–75% range; blue dots: ensemble median), respectively. (b) DJF NAO index in ERA‐20C (red) and the ASF‐20C re‐forecasts (blue). Blue shades show the re‐forecast ensemble distribution, similar to (a).

Figure 1(a) displays DJF‐mean global mean 2 m temperature anomalies over the period 1900–2009 from the ASF‐20C ensemble (shades of blue) started on 1 November each year and from ERA‐20C (red line). Here the blue shading shows the ensemble distribution of the re‐forecast ensemble with the lighter blue shades indicating the full range of the ensemble, the darker shade indicating the interquartile range between the 25th and 75th percentiles and the dark blue dots denoting the ensemble median. As a proxy for estimating the uncertainty of the 20th Century reanalysis data, the orange shading surrounding the red ERA‐20C line shows the temperature range of a ten‐member ensemble of reanalysis generated with an earlier version of ERA‐20C (Poli *et al*., 2015). Although the reanalysis ensemble spread is larger at the beginning of the century reflecting larger uncertainties in the reconstruction of the global mean temperatures in this early period, the overall level of reanalysis ensemble spread of seasonal mean temperature is too small to be realistic; see Poli *et al*. (2015) for discussion.

As can be seen from Figure 1(a), the 110‐year hindcast ensemble captures the verification data very well. In particular, the multi‐decadal fluctuations and the strong warming during the last decades are well reproduced in the forecasts, though the model somewhat underestimates the global cooling period of the 1950s–1980s. A comparison of global land and sea areas separately (not shown) indicates that the underestimation is larger over land but still present over sea, even though the model is forced by prescribed SSTs at its lower boundary.

The NAO, with its strong impact on the weather and climate over the Atlantic–European sector, varies on multiple time‐scales from days to years and decades (Woollings *et al*., 2014). Here we analyse the retrospective seasonal forecast skill of the winter NAO in the ASF‐20C data. We define the reference NAO index as the principal component (PC) of the leading empirical orthogonal function (EOF) of DJF anomalies of geopotential height at 500 hPa (Z500) over the Atlantic sector (90°W–30°E, 30°N–90°N) calculated from the ERA‐20C reanalysis data. The re‐forecast NAO index from ASF‐20C is computed by projecting each ensemble member onto this reference EOF.

**Figure 2:**
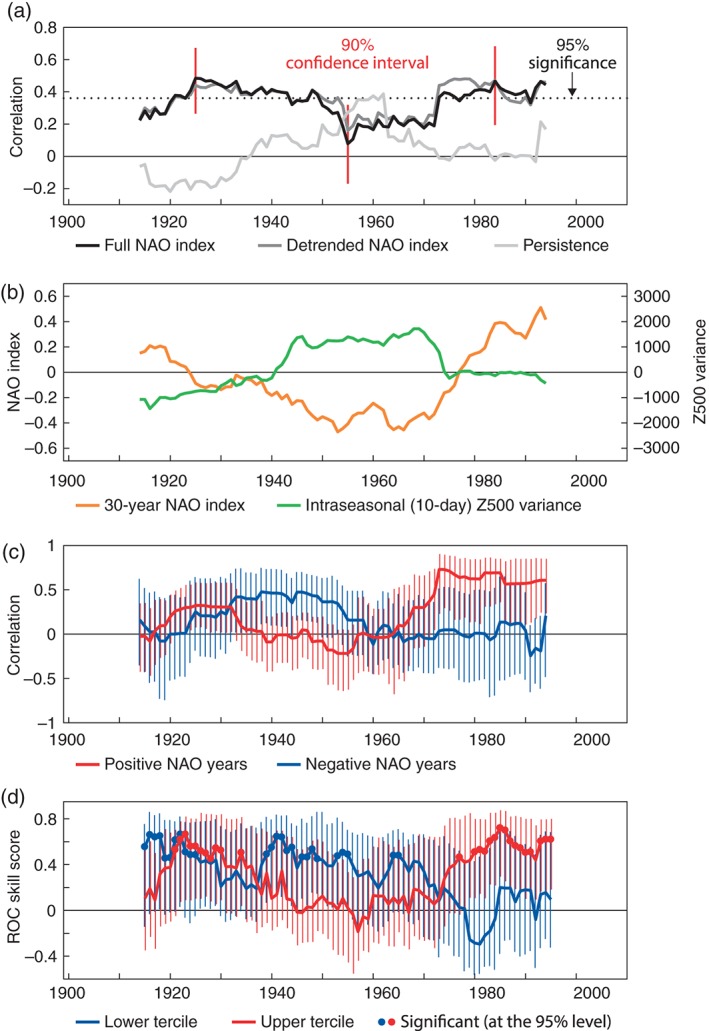
(a) Anomaly correlation coefficient (ACC) of the DJF NAO index between the ensemble mean ASF‐20C and ERA‐20C (black) over the period 1900–2009 computed for moving 30‐year windows by 1 year. Values are plotted at the 15th year of each window. Dark grey: ACC when a linear trend in each 30‐year window has been removed before the computation. Light grey: ACC of a simple statistical forecast using persistence of the November average NAO index. The dotted horizontal line indicates the t‐test 95% significance level of the correlations and the red vertical bars show 90% confidence intervals estimated from bootstrap re‐sampling (1000 times) with replacement for three representative periods. (b) 30‐year running mean filtered DJF NAO index in ERA‐20C (orange) and area‐averaged intraseasonal variance of 10‐day mean Z500 in the Atlantic sector computed from moving 30‐year windows and expressed as anomalies (green), units in m^2^. (c) ACC of the DJF NAO index for years with positive (red) and negative (blue) indices in ERA‐20C computed for moving 30‐year windows. Vertical bars indicate the confidence intervals. (d) ROC Skill Score (ROCSS) of the DJF NAO index being above the upper tercile (red) and below the lower tercile (blue). Dots indicate where the ROCSS is significantly different from zero at the 95% level according to a non‐parametric Mann–Whitney U‐test and vertical bars indicate confidence intervals.

Figure 1(b) shows the NAO index for DJF from the deterministic ERA‐20C (red line) and the ensemble of seasonal hindcasts, in a similar fashion to Figure 1(a). The NAO index from ERA‐20C is in very good agreement with other estimates of the index, for example, the correlation with the Hurrell *et al*. (2003) PC‐based sea‐level pressure (SLP) NAO index is 0.96. The ASF‐20C re‐forecast ensembles include the verifying index for all but three years (1978, 1988 and 2009). Two of these are the most extreme positive and negative NAO winters during the entire 110‐year period. The NAO was in predominantly positive phases at the beginning of the twentieth century and during the last three decades, while the negative phase was more pronounced from the 1940s to the 1970s; see also low‐pass filtered NAO index in Figure 2(b). In the following section we describe and discuss the skill of the forecast model predicting the NAO index throughout the twentieth century.

## 3 Multi‐decadal variability of predictive skill of the NAO

Seasonal forecasts suffer from model biases that can, to first order, be corrected for by computing observed and model anomalies from a long‐term climatology of observations and the model, respectively. The ensemble‐mean anomaly correlation coefficient (ACC) is often used as a simple deterministic measure of interannual forecast skill. Computed over the entire 110‐year forecast period, the ACC for the NAO index is 0.31, which is highly statistically significant (*p*‐value <0.001, using the *t*‐test). The 90% confidence interval for the correlation coefficient of [0.17, 0.45] excludes zero indicating a promising level of overall forecast skill in this system during the twentieth century.

Seasonal forecasts traditionally focus on the more recent decades due to the improved quality of initial data for the atmosphere and ocean. The NAO correlation skill of ASF‐20C during the latest 30‐year period 1980–2009 is 0.44 (*p* = 0.01 with a 90% confidence interval of [0.16, 0.66]). In order (i) to test the impact of reducing the coupled ocean–atmosphere system to an atmosphere‐only system, and (ii) to see the impact of using an atmospheric reanalysis that assimilates not only surface pressure and marine winds but all the range of *in situ*, airborne and satellite data, we have run seasonal hindcasts for 1980–2009 using the same atmospheric forecast system and initialisation but (i) coupled to the Nucleus for European Modelling of the Ocean (NEMO) ocean model (Molteni *et al*., 2011), and (ii) initialised using ERA‐Interim (Dee *et al*., 2011). The coupled re‐forecasts result in an ACC for the NAO of 0.48 indicating a rather constant level of correlation skill regardless of whether prescribed or interactive SSTs are used. The uncoupled re‐forecasts using ERA‐Interim rather than ERA‐20C for the initialisation of the atmosphere result in a comparable level of ACC (0.40). The fact that using ERA‐20C for the initialisation of the atmosphere leads to very similar results as using ERA‐Interim for the initialisation thus enhances the confidence in using ERA‐20C for earlier periods.

In order to diagnose the multi‐decadal variability of the NAO forecast skill throughout the century, we now analyse the evolution of the ACC between ERA‐20C and the ensemble mean of ASF‐20C during the 110‐year hindcast period. To compare with previous hindcast experiments, which have typically been performed over periods of 20–30 years, the ACC has been calculated between the forecast anomalies and the corresponding verifying anomalies for a moving 30‐year window. The ACC for the NAO forecasts for each 30‐year period is shown in Figure 2(a), and exhibits marked variability on multi‐decadal time‐scales across the 110‐year period. While the estimated ACCs are positive throughout the entire century, there are coherent groups of multiple decades where our analysis suggests that the skill over the different 30‐year periods exceeds the 95% significance level of a *t*‐test. These include the years centred around the mid‐1920s to mid‐1940s and from the mid‐1970s onwards. The skill is lower, though still positive, for all of the 30‐year periods centred between the early 1950s and mid‐1970s.

How significant are these variations in forecast skill though? The *t*‐test statistics and the confidence intervals of the estimated correlation coefficients (Figure 2(a)) indicate that it is reasonable to assume there is skill in the ensemble‐mean forecast of the NAO at least for the earlier and late periods. The skill of the forecasts appears to weaken in the middle part of the century, but the statistical evidence for a significant difference between the periods is, however, not overwhelming. Figure 2(a) displays the 90% confidence intervals for the three non‐overlapping periods 1911–1940 (representative for the early high‐skill period), 1941–1970 (representative for the middle low‐skill period) and 1979–2008 (representative of the late high‐skill period). Due to sampling uncertainty, the confidence intervals are relatively large and partly overlap, though these still give an indication of a difference in skill during the different decades.

However, in order to show that this decadal variation in skill is not just a statistical artefact, in the following we present supportive evidence that these multi‐decadal variations in skill (with a moderate level of statistical significance) co‐vary strongly with statistics of the general circulation itself. The combination of a marginally significant time series of skill correlated with various diagnostics of the circulation will lead us to hypothesise the existence of genuine multi‐decadal fluctuations in the level of seasonal forecast skill of the NAO.

### 3.1 *Linkages to the NAO phase and amplitude*

Comparing the 30‐year correlation skill from Figure 2(a) with the 30‐year running mean NAO index in Figure 2(b) reveals that, in general, periods of significant forecast skill coincide with periods when the 30‐year averaged NAO index is positive. For example, the mid‐century decades of low correlation skill agree with periods when the NAO was in a strongly negative phase. At first glance this suggests that the model is struggling to predict the circulation in seasons with negative NAO. To test this, we recalculated the correlation coefficient in each 30‐year period for only the positive and negative NAO winters; these are shown in Figure 2(c). The vertical bars indicate the confidence intervals, as estimated by bootstrap resampling from each 30‐year period with replacement. The correlation skill from the early 1970s onwards seems to come primarily from the skilful prediction of positive NAO years, whereas in earlier periods negative NAO years appear to contribute at least as much, if not more, to the correlation skill. We cannot therefore conclude that the model is unable to skilfully predict negative NAO winters in general.

It is useful to compare the ASF‐20C forecasts with a benchmark statistical forecast in order to gauge the value of using the dynamical model based on physical principles. Here we construct a simple persistence forecast which targets the same DJF season as ASF‐20C. As a predictor we use the monthly mean NAO index of ERA‐20C from the previous November. The resulting ACC skill is plotted in Figure 2(a) (light grey line) for each 30‐year period, following the same format as the ASF‐20C. The correlation skill of the persistence forecast is lower than that of the ASF‐20C and not significant for most of the century, except for a period centred around 1960, in which the correlation reaches a peak. This period of maximum skill in the statistical reference forecast coincides with the drop in skill seen in ASF‐20C, and the period of generally low NAO indices as seen in Figure 2(b). If such negative NAO states are related to tendencies for longer persistence time‐scales and the dynamical forecast model has deficiencies in simulating such persistent situations of negative NAO flow patterns in the atmosphere, then it is plausible that the positive forecast skill using persistence and the drop in skill of the dynamical forecast model could be related. However, exploratory analysis of the hypothesis by looking at these metrics across all years individually, rather than at 30‐year statistics, did not reveal any clear relationship.

The ACC is a deterministic measure of forecast skill as it is based on one deterministic forecast, the ensemble mean forecast. However, the full ensemble with its 51 forecast members offers a deeper insight into the probabilistic nature of NAO forecast skill by constructing probability forecasts for various values of the NAO index (i.e. Figure 1(b)). In the following we analyse the Receiver Operating Characteristic (ROC) skill score, ROCSS (e.g. Mason and Graham, 2002; Jolliffe and Stephenson, 2003), of the NAO forecasts as two metrics of probabilistic skill.

The ROCSS measures the ability of a probabilistic forecast to detect the occurrence of a dichotomous event, for example a variable falling above or below a certain threshold, and encompasses hit and false alarm rates measured through a range of thresholds. As such the ROCSS is a categorical score which requires the definition of the events or category. The score is calculated with respect to a reference forecast of climatological frequency, such that ROCSS = 1 indicates that the forecast system can perfectly discriminate events from non‐events, whilst ROCSS = 0 indicates that a system does not offer improvement over simply using the climatological frequency as the event forecast probability. We have chosen the ROCSS because it is a commonly used skill score in the verification of operational seasonal forecasts.

**Figure 3:**
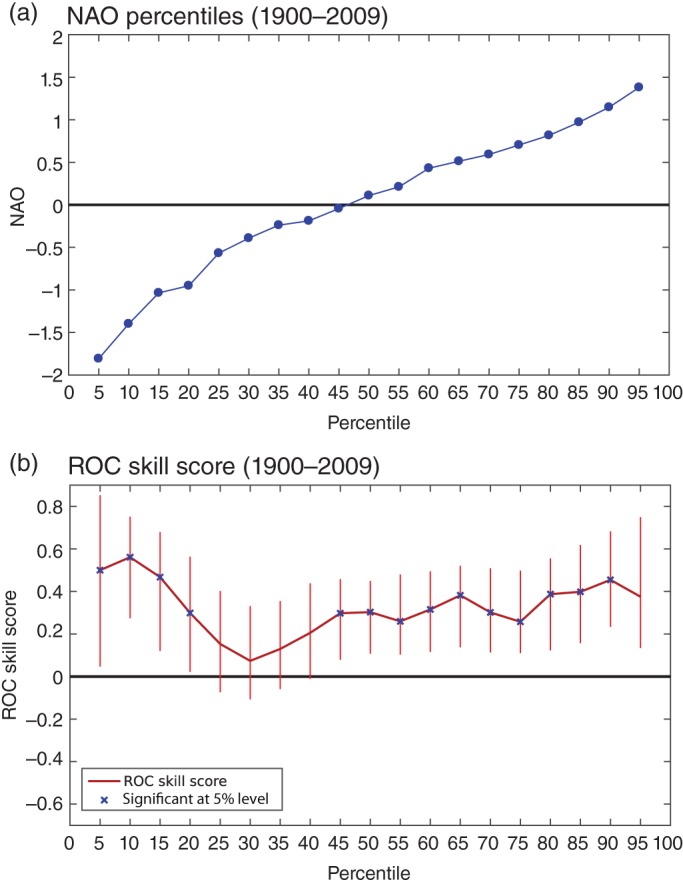
(a) Cumulative distribution (percentiles) of the DJF NAO index in ERA‐20C computed from all data in the period 1900–2009. (b) ROCSS of predicting the DJF NAO index during the period 1900–2009 for varying event thresholds from the 5th to the 95th percentiles of the distribution. Blue crosses indicate the significance according to a non‐parametric Mann–Whitney U‐test and vertical bars indicate confidence intervals.

From the entire 110 years of NAO forecasts we can construct a climatology of the forecast index and compute the percentiles of the distribution in steps of 5% (Figure 3(a)). We then use all of these percentiles separately as the thresholds for defining binary events above these thresholds in the computation of the ROCSS (Figure 3(b)). The median, upper and lower tercile events, for example, correspond to the 50th, 67th and 33rd percentiles. The significance of the ROCSS was calculated using the non‐parametric Mann–Whitney *U*‐test (following Mason and Graham, 2002) and is indicated by the blue crosses in Figure 3(b). The confidence intervals were calculated separately using a bootstrap resampling, performed 1000 times with replacement, essentially giving two estimates of the statistical significance for the ROC skill scores. As can be seen, the probabilistic forecasts of the NAO index being below the 10th percentile (corresponding to an NAO index of approx. −1.4) appear to have the highest ROCSS of all thresholds. As can be also seen from Figure 3(b), over the whole hindcast period 1900–2009, the ROCSS for predicting NAO indices for all ranges of NAO percentile threshold is positive. Moreover, the ROCSS is significantly positive at all thresholds apart from those between the 20th and 45th percentiles, corresponding to NAO values between −1 and 0. In this sense, the model does not perform as well at predicting weak negative NAO winters as for predicting positive NAO index winters. However, the model performs remarkably well at predicting strong negative NAO winters, indicating that the model clearly does not struggle with negative NAO winters of all magnitudes.

**Figure 4:**
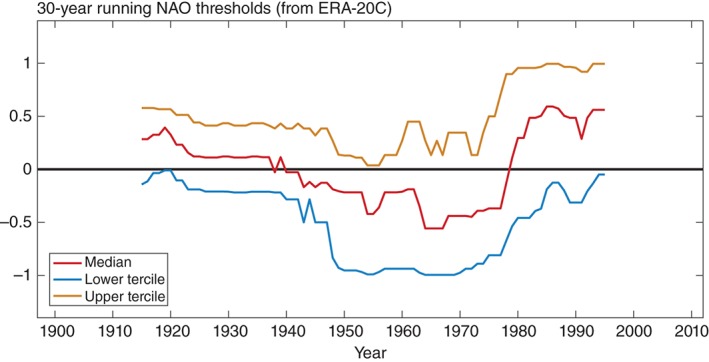
Absolute thresholds of the DJF NAO index in ERA‐20C corresponding to the lower tercile (blue), upper tercile (yellow) and median (red) during each moving 30‐year window.

We analyse how the skill of the probabilistic NAO forecasts varies over the entire hindcast period by calculating the ROCSS for the upper and lower tercile events over moving 30‐year periods, shown in Figure 2(d). Here, upper (lower) tercile events correspond to forecasts within the upper (lower) third of the corresponding 30‐year NAO index distribution. The vertical bars show the confidence interval estimated through a bootstrap approach while the blue and red dots indicate significance according to a Mann–Whitney *U*‐test, as in Figure 3(b). In the most recent period, from the mid‐1970s onwards, the model demonstrates impressive significant skill in forecasting the probabilities of upper‐tercile NAO events, which is consistent with the skill of the positive NAO ensemble mean forecasts in this period (i.e. Figure 2(c)). In the earlier periods, up to around 1950, the model generally produces skilful probabilistic forecasts for both the upper and lower terciles. The overall NAO skill in the earlier period thus stems from skilfully predicting a spectrum of NAO events, which is also highlighted in the ensemble mean correlation skill in Figures 2(a) and (c). During the period centred between the early 1950s and early 1970s the model has no significant ensemble mean correlation skill (Figure 2(a)). However, the model does demonstrate significant skill for the lower‐tercile events during part of the middle of the twentieth century (blue curve in Figure 2(d)). In this period, the lower‐tercile events correspond to winters with a strongly negative NAO index (that is less than about −1, see tercile thresholds for each period in Figure 4), which is consistent with the finding shown in Figure 3(b) that the model is skilful for strong negative NAO events (up to the 20th percentile) over the entire hindcast period.

### 3.2 *Trends as a potential source of predictive skill?*

The strong positive trend in the NAO from the mid 1970s onwards (Figure 2(b)) synchronising with significantly positive forecast skill during this period raises the question whether trends in the data are the primary source of forecast skill. In order to test this hypothesis, we have removed the linear trend in each of the moving 30‐year windows and computed the ACC of the detrended anomalies. The result is shown with the dark grey line in Figure 2(a). This detrending, however, has little effect on the multi‐decadal variability in forecast skill. The level of skill is overall slightly increased for the detrended data suggesting that any existing trends may even act to reduce the skill.

### 3.3 *Relationship with other indices of the general circulation*

**Figure 5:**
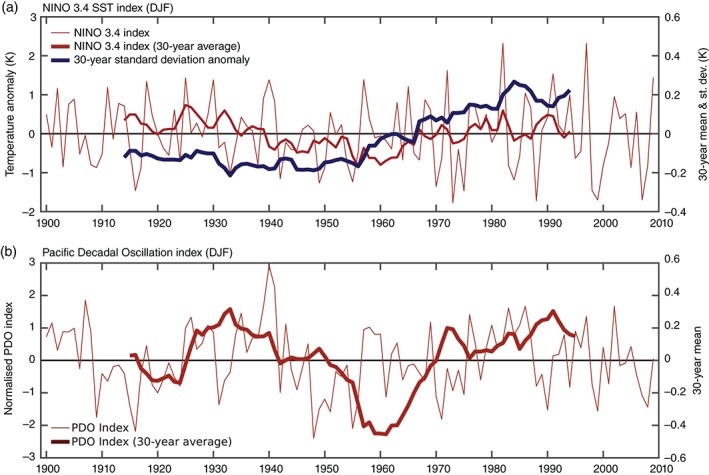
(a) Time series of DJF‐mean central tropical Pacific SST anomalies of annual (light blue) and 30‐year average (dark blue) data and their variability during moving 30‐year windows (black line) in HadISST (Rayner et al., 2003) for the Niño 3.4 index. (b) The annual and 30‐year mean PDO index (from https://www.ncdc.noaa.gov/teleconnections/pdo/).

The mid‐century period during which the NAO hindcast skill is relatively small has been proven to be difficult to predict in previous studies (Müller *et al*., 2005; Shi *et al*., 2015). Greatbatch and Jung (2007) noted that the low level of NAO forecast skill during the period 1962–1981 corresponded to a period of weak seasonal diabatic heating anomalies in the tropical Pacific. The lower variance of the tropical Pacific SSTs during this period might explain the lack of predictable skill in the NAO hindcast compared to the more recent period (i.e. from the 1980s onwards), particularly as Scaife *et al*. (2014) find that much of their NAO forecast skill is associated with the response to different phases of El Niño. The Niño3.4 central tropical Pacific SST index used in the model is shown in Figure 5(a). However, the skill of the earlier period coincides with general low levels of ENSO activity in terms of variability of tropical Pacific SST (dark curve in Figure 5(a)) so that we cannot explain the variability in skill over the hindcast period through El Niño variability alone.

The forecast skill for each of the 30‐year periods does seem to be related to the dominant phase of El Niño (Figure 5(a)), with periods of positive SST anomalies in the central tropical Pacific being coincident with the early and late periods of strong NAO skill in the hindcasts. This is also the case for the Pacific Decadal Oscillation (PDO: e.g. Mantua *et al*., 1997; Minobe, 1997), shown in Figure 5(b), which is in a positive phase during periods when the hindcasts are most skilful. Extratropical circulation primarily forces the PDO (Newman *et al*., 2016) and as such this may indicate that different circulation patterns in the extratropical North Pacific have an influence on predictability in the North Atlantic sector. This certainly merits further investigation and is something we are actively studying.

Whether the trends in the NAO towards a positive polarity in the later periods can be understood as the remotely forced atmospheric response to the progressive warming of the tropical Indian Ocean since the 1950s, as suggested by Hoerling *et al*. (2001, 2004), and if there is a possible link to the NAO forecast skill, remains another hypothesis for future research.

## 4 The use of seasonal forecasts to increase confidence in extreme event attribution

Extreme weather and climate events affect many aspects of our society. Understanding and predicting extremes is one of the Grand Science Challenges of the World Climate Research Programme (WCRP) and includes the question of how extremes are likely to vary under the impact of changing climate. The emerging field of probabilistic event attribution tries to provide answers to the question of whether human activity has increased the risk of occurrence of such events; see the recently published consensus report by the National Academies of Sciences, Engineering and Medicine (2016) for a review of the science of attribution. The report identified as a priority research need the development of links to an integrated weather‐to‐climate forecasting effort on a range of time‐scales in order to promote reliable assessments of the performance of event attribution systems. As a first step towards such an integrated attribution framework, we propose the use of our new ASF‐20C seasonal hindcast ensemble to provide attribution statements on extreme seasonal climate events with quantitative estimates of their reliability.

An event attribution statement is often expressed in terms of probabilities that an extreme event occurs (i) under current conditions that include anthropogenic contributions, and (ii) under hypothetical conditions that are not affected by anthropogenic forcings. While (i) describes the factual world as it is experienced, (ii) refers to a counterfactual world that does not exist but can be approximated using either observations from the past, weather and climate models or a combination of both. The so‐called Fraction of Attributable Risk (FAR: Stott *et al*., 2004; not to be mistaken with the False Alarm Rate) estimates how the anthropogenic influence has altered the risk of an extreme event:

$$\text{FAR}=\left(P_{\text{FACT}}-P_{\text{COUNT}}\right)/P_{\text{FACT}}$$

where P_FACT_ denotes the probability incurring under current factual conditions (i) and P_COUNT_ denoting the probability of a counterfactual world (ii). There are different ways of estimating P_FACT_ and P_COUNT_ based on observations of past and recent periods or on simulations with climate models where the anthropogenic radiative forcings can be controlled (Shepherd, 2016; Stott *et al*., 2016).

One popular approach (e.g. Pall *et al*., 2011; Schaller *et al*., 2016) includes running very large ensembles of climate simulations with an atmosphere‐only climate model to estimate P_FACT_ and P_COUNT_. However, all climate models are prone to biases and unreliability in their estimates, especially for probabilities of rare events. In order to build more confidence in these estimates and attribution statements derived from model‐based probabilities, it is essential that the used probabilities are reliable (Christidis *et al*., 2013; Lott *et al*., 2013). While FAR‐based attribution statements are increasingly being issued with an uncertainty range based on ensembles of model simulations, so far information about the reliability of these model‐based probabilities in the first place has rarely been included in attribution statements. We argue that such reliability information is crucial to enhance trust in the science of attribution. By reliability we mean a very specific and quantifiable statistical characteristic of probabilities: a probabilistic system is called reliable if the model probabilities for a certain event equal, within some error bounds, the observed frequencies of occurrence of the event; see Weisheimer and Palmer (2014) for a discussion of this concept for seasonal forecasts.

A recent study by Bellprat and Doblas‐Reyes (2016) highlighted the importance of reliable probabilities for the attributable risk of extreme weather and climate events. Using the idealised framework of a conceptual model, it showed that low reliability introduces a systematic effect resulting in an overestimation of the absolute FAR values, leading to overconfident attribution statements.

We propose to estimate forecast model reliability using our ensemble of ASF‐20C. This approach offers the following advantages. Firstly, seasonal forecasts are model simulations that can be statistically verified against observations (or reanalyses) for every forecast made, which is an essential criterion for building trust in model‐derived probabilities. Secondly, seasonal forecasts such as ASF‐20C use a state‐of‐the‐art dynamical global atmospheric model to estimate the probability of the state of the atmosphere throughout a season. Both seasonal forecast and extreme event attribution studies use essentially the same class of climate models. Therefore, results from the reliability analysis of the seasonal forecasts are indicative of the reliability of other models' probabilities if we assume that the fundamental physical processes leading to the extremes are well represented in these models. Finally, the availability of more than 100 years of seasonal hindcasts makes it possible to estimate the reliability of its model probabilities both for an atmosphere that has experienced anthropogenic forcings and influences up to 2010 (a surrogate for P_FACT_) and for an atmosphere that was, to first‐order approximation, free of large anthropogenic forcings near the beginning of the twentieth century (a surrogate for P_COUNT_). ASF‐20C thus provides the opportunity to complement estimates of FAR from the long hindcast series with quantitative measures of reliability of the probabilities P_FACT_ and P_COUNT_. The use of pairs of reliability diagrams has also been recommended to provide observational estimates of the accuracy in FAR in a recent study by Lott and Stott (2016).

**Figure 6:**
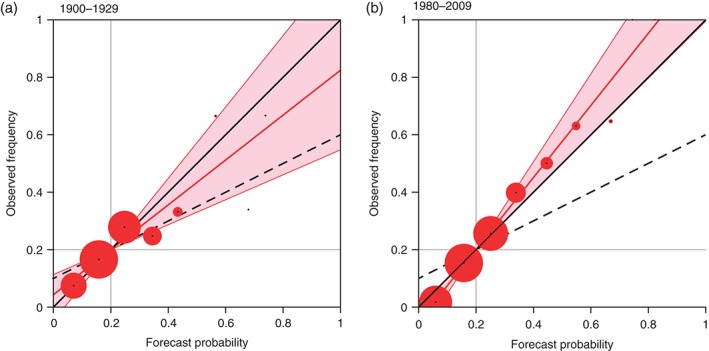
Reliability diagrams for winter temperatures over Southern European land points falling within the lowest quintile for a period at (a) the beginning and (b) the end of the twentieth century estimated from ASF‐20C using ERA‐20C as verification. The red line is the weighted linear regression of all data points with the red shaded area indicating the uncertainty of the regression line, see Weisheimer and Palmer (2014) for details.

To demonstrate how the reliability concept is proposed to complement attribution statements made using the FAR, let us consider the example of cold winters over Southern European land areas below the lower quintile (20th percentile). Here, the fundamental analysis tool is the reliability diagram; see Figure 6 for the example event. The two diagrams in Figure 6 are estimates of model reliability for a period near the beginning of the century from 1900 to 1929 in Figure 6(a) and for the most recent period 1980–2009 in Figure 6(b) using ERA‐20C as verification. The red dots show a range of binned model probabilities (horizontal axis) and their corresponding observed frequency of occurrence (vertical axis). The size of the dots is proportional to the number of data points in each probability bin. Perfectly reliable probabilities would lie on or near the diagonal, while probabilities with little or no reliability would be scattered around a flat distribution indicating little or no link between the model probabilities and the observed frequencies. Weisheimer and Palmer (2014) suggested a simple categorisation using the slope of a weighted regression (red line) between the model probabilities and observed frequencies and its uncertainty range (light red area around the regression line) as a measure of reliability (see also Murphy and Wilks (1998)). In our example case of cold Southern European winters in Figure 6 it can be seen that the seasonal re‐forecasts are very reliable during both periods. The uncertainty margins of the regression lines include the perfect reliability diagonal. The calculation of the FAR for the event of temperature being within the lower quintile of the recent period results in FAR = −1.2 corresponding to a 55% decrease in risk of the cold winters from the beginning of the century to current conditions. The reliability analysis suggests that our confidence in P_COUNT_ and P_FACT_ is very high and the derived FAR statement can subsequently be considered trustworthy.

In cases where the reliability of the probabilities is moderate, the FAR statements should be made with caution and perhaps be corrected. If, however, the reliability of the model probabilities is poor, as is often the case for precipitation and in general for coupled atmosphere–ocean forecasts where the SSTs are predicted rather than prescribed, then the resulting FARs are unreliable, likely to be overstated and should not be considered trustworthy. Other examples of reliable and unreliable forecast events for the factual world with anthropogenic impact on the atmosphere are presented in Weisheimer and Palmer (2014).

## 5 Summary and conclusions

The new global seasonal retrospective ensemble forecasting dataset ASF‐20C based on ECMWF's atmospheric model covers the boreal winter seasons of the entire twentieth century from 1900 to 2010. The unprecedented size of its hindcast in terms of the period covered and the number of 51 ensemble members allow for a thorough inspection of the robustness of seasonal forecast skill estimates and their variability on a time‐scale much longer than in previous studies.

In the first part of the article we have demonstrated that while the ASF‐20C hindcasts show positive and significant interannual correlation skill of the winter North Atlantic Oscillation for the entire forecast period, the predictive skill of the NAO is exposed to some multi‐decadal variability. One motivation for the current research was to attempt to replicate the previously reported variations in skill using a model not used in these earlier studies. We also wanted to extend the seasonal hindcast datasets backwards to the beginning of the twentieth century, in order to assess whether the variation of skill with decade was monotonic. We are able to confirm relatively low levels of skill during the period of the 1950s and 1960s as found by previous studies (Müller *et al*., 2005; Shi *et al*., 2015). Interestingly, the skill appears to increase again for earlier start dates suggesting poor initial data was not the cause of these relatively low scores. Using confidence intervals and *p*‐values, we find that these differences in skill are only marginally statistically significant. However, there is also evidence for a meteorological explanation of non‐stationary predictability. As a way to either confirm or refute the hypothesis that these variations in skill are not a statistical artefact, we have assessed the extent to which they correlate with variations in the general circulation itself. We find strong correlations between time series of NAO skill, and low‐frequency time series of the NAO itself, over the whole twentieth century. There is no obvious statistical reason why these two time series should be correlated. We also find other correlations between skill and decadal time‐scale diagnostics of the PDO and ENSO. These correlations with the general circulation suggest that the decadal variations in NAO forecast skill, otherwise marginally significant, are indeed genuine.

These findings are in agreement with results from statistical hindcasts of the winter NAO during 1900–2001, which use, as predictors, the near‐surface temperature during the preceding months over the Northern Hemisphere sub‐polar regions (Fletcher and Saunders, 2006). These authors conclude that the hindcast skill is non‐stationary and that the highest positive skill is observed during the early and late twentieth century.

Although the periods of high (low) levels of NAO skill seem to coincide with periods of high (low) levels of the NAO index, no general evidence was found that the forecast model cannot skilfully predict negative NAO winters. Rather, our analysis suggests that probabilistic forecasts for strong negative and all ranges of positive NAO indices were highly skilful indeed. The model does not perform as well for weak negative NAO events with an index between −1 and 0 though, which is a curious feature and merits further attention. The overall skill in the first half of the twentieth century stems from skilfully predicting a wide spectrum of NAO events. Our findings agree with results reported in Müller *et al*. (2005) about a weak relationship between the NAO amplitude and NAO forecast skill based on multi‐model seasonal forecasts of the four last decades of the twentieth century.

Trends in the NAO index were found to have a negligible impact on the skill.

During the mid‐century period of low skill, the DJF NAO exhibits remarkable persistence from the November NAO, as shown in Figure 2(a). This level of NAO persistence is not observed during the rest of the hindcast period. The decades of high NAO persistence from the 1940s to the 1970s coincide with periods of enhanced intraseasonal variability of Z500 over the Atlantic sector (Rennert and Wallace, 2009) as shown in Figure 2(b). These findings are consistent with the hypothesis that upper‐level Rossby wave‐breaking events occur more frequently during periods of negative NAO, than during periods of positive NAO (Benedict *et al*., 2004; Woollings *et al*., 2008).

Further study is needed to understand why there appears to be such low skill in the mid‐century period. It is not possible to conclude whether this is due to a flow‐dependent nonlinear model error, which prevents the model from being able to simulate extended periods of a relatively stable flow with persistent negative NAO indices, or whether the intrinsic predictability of the atmosphere was lower than during other periods of the twentieth century. It becomes clear, however, that the mid‐century decades stand out as an important period on which to test the performance of future seasonal forecast systems. Achieving good forecast skill for the more recent decades with predominantly positive NAO indices is not sufficient to guarantee similarly good performance for periods with a stronger tendency for negative NAO states that might possibly occur in the future again.

The second part of the article discussed another area of potential application of the ASF‐20C dataset, the use of reliability estimates of ASF‐20C seasonal forecast probabilities to increase the confidence in statements of extreme weather and climate event attribution to anthropogenic climate change. We see the proposed use of seasonal forecasts as a first step towards developing synergies with weather and climate forecasting in line with the recently defined future research priorities for the science of extreme event attribution. Probabilities derived from seasonal retrospective forecasts have the advantage that they can be verified against observations using the concept of statistical reliability. For the example case of very cold winters over Southern Europe the seasonal forecast reliability analysis suggests that our confidence in the attribution probabilities P_COUNT_ and P_FACT_ is very high and that the derived fraction of attributable risk statement could be considered trustworthy. For cases, however, where the reliability of the model probabilities is poor, the attribution statements should not be considered trustworthy because the FAR is unreliable and likely to be overstated.

It is planned for future work to demonstrate how reliability diagrams are not only useful tools to evaluate the relationship between modelled and observed probabilities but at the same time offer a straightforward way to calibrate any unreliable system so that its probabilities become reliable (Palmer *et al*., 2008; Matsueda *et al*., 2016).

The analysis of multi‐decadal variability in forecast skill of the NAO and the demonstration of the potential use of the long hindcast data for improved extreme weather and climate event attribution statements are just two examples of what could be studied in such a long climate forecast dataset. In the future we intend to analyse a wider range of atmospheric variability components and their predictability, including atmospheric teleconnection patterns linked to ENSO and stratospheric circulation.

The presented seasonal forecasting system requires the provision of sea‐surface temperatures as a lower boundary to the atmosphere. As such the forecasts are purely atmospheric forecasts assuming perfect knowledge of the oceanic boundary conditions. With the production of a new twentieth century reanalysis of the coupled ocean–atmosphere system (Coupled ECMWF ReAnalysis (CERA): Laloyaux and Dee, 2015) well under way, similarly long coupled atmosphere–ocean seasonal re‐forecasts are now becoming feasible.
